# A Model of BGA Thermal Fatigue Life Prediction Considering Load Sequence Effects

**DOI:** 10.3390/ma9100860

**Published:** 2016-10-24

**Authors:** Weiwei Hu, Yaqiu Li, Yufeng Sun, Ali Mosleh

**Affiliations:** 1 Reliability and System Engineering School, Beihang University, Haidian District, Beijing 100191, China; hww@buaa.edu.cn (W.H.); syf@buaa.edu.cn (Y.S.); 2 B. John Garrick Institute for the Risk Sciences, University of California, Los Angeles, CA 90095, USA; mosleh@umd.edu

**Keywords:** BGA, life prediction, load sequence effects, crack growth, damage accumulation, resistance strain

## Abstract

Accurate testing history data is necessary for all fatigue life prediction approaches, but such data is always deficient especially for the microelectronic devices. Additionally, the sequence of the individual load cycle plays an important role in physical fatigue damage. However, most of the existing models based on the linear damage accumulation rule ignore the sequence effects. This paper proposes a thermal fatigue life prediction model for ball grid array (BGA) packages to take into consideration the load sequence effects. For the purpose of improving the availability and accessibility of testing data, a new failure criterion is discussed and verified by simulation and experimentation. The consequences for the fatigue underlying sequence load conditions are shown.

## 1. Introduction

Solder joint interconnects serve as electrical connections and mechanical bonds between components and the substrate [1]. Small package size trends dictate leadless solder ball grid arrays (BGA) becoming the mainstream, as a result BGA package reliability has been put at the forefront of research issue.

Fatigue life prediction models have been investigated based on a diverse range of solder joints, all the models require some specific geometry and material related information to establish constitutive equations [2]. Some experts concentrate on the damage mechanisms and conditions, for example: electronic products may experience thermal cycle, power, shock, and vibration during the whole life cycle. Literature [3] shows three quarters of BGA fatigue failures are the result of thermal and vibration load, and researchers developed models by associating the stress-strain data under certain load with damage situation. For now, most of the models applied in engineering follow the Miner linear damage accumulation rule for its convenience. However, some researchers believe that the variation of load conditions also has an effect on the BGA products failure time [4], study [5] indicates that the linear cumulative damage law cannot conform to the experiments well. So they focus on the sequence influence of individual cycles in physical fatigue damage accumulation. A study by Okuyama et al. [6] demonstrates that the load conditions of thermal to vibration are harsher than in reverse. Fatemi and Yang [7] provided an overview on cumulative fatigue damage and life prediction, but these models do not have good practicability.

This paper tries to propose a life prediction model for BGA packages that takes load sequence influence into consideration that is considered more appropriate for application. Actually, it is extremely difficult to monitor and track the initiation and propagation of fatigue cracks of BGA packages, consequently we define electrical resistance strain, whose value is equal to ratio of the resistance variation to initial electrical resistance, and can reflect the quantitative relationship between the extent of solder joint resistance variation and crack expansion as an alternative failure criteria for measuring the physical fatigue damage.

## 2. Thermal Fatigue Life Prediction Model for BGA

### 2.1. Model Assumption

Failure occurs when the fatigue crack of BGA solders reaches a critical length $a_{C}$; the fatigue failure process of BGA solder joints can be divided into initial crack stage and crack propagation stage, the fatigue life of solder joints can be described as Equation (1) shows,

(1) $$N_{f}=N_{o}+N_{p},$$

where $N_{f}$ means the whole fatigue lifetime of solder joints, $N_{o}$ represents the lifetime of initial cracks, namely the cycles to crack initiation, and $N_{p}$ represents the lifetime of the propagation stage, respectively [8]; assuming that cyclic thermal load conditions in the initial crack stage may affect the life of solder joints.

**Figure 1:**
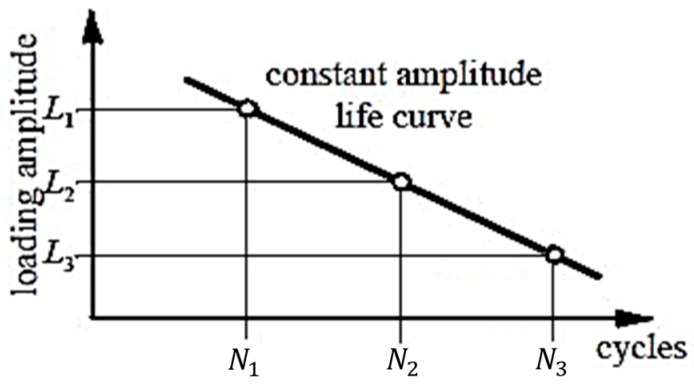
Scheme of different loading amplitude versus solder lifetime cycles.

Several studies [9,10] indicate that cyclic thermal load conditions result in material hardening or softening, which may change the material hardness of solder joints. Bao [11,12] points out that when the recovery resistance of material is the same, the elastic modulus is proportional to the square root of the material hardness, therefore the elastic modulus of solder joints may be influenced and subsequently the stress situation is affected. In addition, loads with different amplitudes have different effects on inner defect of solder joints [13], Figure 1 shows that low loading amplitude can help release the inner stress of defects and extend solder joints fatigue life. On the contrary, high loading conditions may promote the expansion of defects and shorten their lifetime. $L_{1},L_{2},L_{3}$ in this figure represent three loading conditions and $N_{1},N_{2},N_{3}$ are their corresponding fatigue cycles, respectively.

Assuming that the load condition effects only exist in initial crack stage.

Abundant tests [14] show that cyclic softening or hardening situation will be stable within a short time, and the lifetime of the initial crack stage is much shorter than that of propagation stage (nearly 10%–20%). Therefore, we suppose that the load condition effects only exist in initial crack stage to simplify the model.

### 2.2. Base Model for BGA Fatigue Life

According to the Darveaux model [15], the relation between crack growth and final failure time can be described as follow,

(2) $$N_{o}=k_{1}{\left(\Delta W_{ave}\right)}^{k_{2}},$$

(3) $$\frac{da}{dN}=k_{3}{\left(\Delta W_{ave}\right)}^{k_{4}},$$

(4) $$N_{f}=N_{0}+\frac{a_{C}}{da/dN},$$

in which $\Delta W_{ave}$ is the incremental viscoplastic strain energy density, $\frac{da}{dN}$ is the crack growth rate and $k_{1}\sim k_{4}$ are correlation coefficients of material properties.

**Figure 2:**
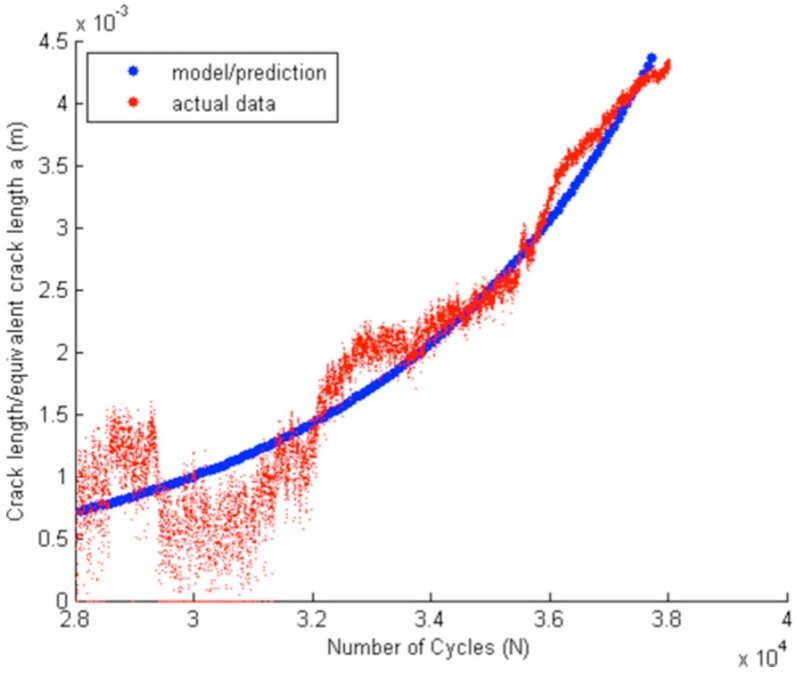
Relationship between the crack length and cycle number in BGA shearing test.

The Darveaux model has been proven to be available in engineering applications, Equation (3) indicates this model suppose that the crack growth rate is constant, which can be linear fitted by test data. However, research [16] demonstrates that crack growth rate increases with the increasing number of cycles in propagation stage, and the increasing trend is similar to exponential growth, as Figure 2 shows.

Hence we improve the Darveaux model with the Paris formula, which is widely used in describing the crack propagation stage, as Equation (5) shows.

(5) $$\frac{da}{dN}=C{\left(\Delta K\right)}^{n}；\Delta K=\Delta \sigma Y\sqrt{\pi a}.$$

where *C* and *n* are material constants, ΔK means amplitude of stress intensity factor, Δσ represents the differences between the maximum stress value and minimum stress value under certain load conditions, Y means the geometry influence function whose value is related to the sample size and load form, in our model we set Y = 1 for simplification [17].

Transforming Equation (5) and integrating from an initial crack length to a variable crack length gives ($N=0,\;a_{0}=0$)

(6) $$a^{-\frac{n}{2}}da=C\Delta \sigma^{n}\pi^{\frac{n}{2}}dN\Rightarrow a={\left(\frac{2-n}{2}C\pi^{\frac{n}{2}}\right)}^{\frac{2}{2-n}}\Delta \sigma^{\frac{2n}{2-n}}N^{\frac{2}{2-n}},$$

take a derivative with respect to cycle number,

(7) $$\frac{da}{dN}=\frac{2}{2-n}{\left(\frac{2-n}{2}C\pi^{\frac{n}{2}}\right)}^{\frac{2}{2-n}}\Delta \sigma^{\frac{2n}{2-n}}N^{\frac{n}{2-n}},$$

let $\alpha =\frac{2}{2-n}{\left(\frac{2-n}{2}C\pi^{\frac{n}{2}}\right)}^{\frac{2}{2-n}}\Delta \sigma^{\frac{2n}{2-n}}$, $\beta =\frac{n}{2-n}$ and we get,

(8) $$\frac{da}{dN}=\alpha N^{\beta },$$

where α is related to thermal cyclic stress condition Δσ in crack propagation stage, thus it can be called in-time load factor. β is denoted as the exponential increasing trend of crack growth ratio, according to the assumption 4 the value of β is related to the stress condition in initial crack stage. When β=0 and da/dN=α is constant, our model coincides with the Darveaux model.

Integrating Equation (8) from zero to critical crack length $a_{C}$ gives equation of $N_{p}$,

(9) $$N_{p}={\left(\frac{\beta +1}{\alpha }\cdot a_{C}\right)}^{1/\left(\beta +1\right)},$$

thus $N_{f}$ can be refined as

(10) $$N_{f}=N_{o}+N_{p}=k_{1}{\left(\Delta W_{ave}\right)}^{k_{2}}+{\left(\frac{\beta +1}{\alpha }\cdot a_{C}\right)}^{1/\left(\beta +1\right)}.$$

### 2.3. Load Sequence Effects in Model

In daily engineering practice, load sequence effects are often ignored and variable amplitude fatigue life are calculated using the linear damage accumulation rule, but the results rarely coincide with experimentally determined data [18]. With the help of α and β in the base model, we are able to consider the load sequence effects in thermal fatigue life prediction of BGA solder joints.

There are two kinds of thermal cyclic load conditions: $\Omega_{\;1}$ and $\Omega_{\;2}$, based on Equation (7) the instantaneous crack growth rates of the sample are,

(11) $$\Omega_{\;i}:da_{i}/dN_{i}=\alpha_{i}N_{i}^{\beta_{i}}，i=1,2,$$

when the crack length $a=a_{1}=a_{2}$, integrating Equation (11) gives

(12) $$a_{i}=\frac{\alpha_{i}}{\beta_{i}+1}N_{i}^{\beta_{i}+1}，i=1,2,$$

combine these two equations together, relation formula between *N*_1_ and *N*_2_ is given

(13) $$N_{1}={\left[\frac{\alpha_{2}(\beta_{1}+1)}{\alpha_{1}(\beta_{2}+1)}\right]}^{\frac{1}{\beta_{1}+1}}\cdot N_{2}^{\frac{\beta_{2}+1}{\beta_{1}+1}}.$$

Assuming that the BGA solder joint sample has experienced $N_{1}=n_{1}$ cycles since passed initial crack stage in $\Omega_{1}$, then the thermal cyclic condition is transformed rapidly into $\Omega_{2}$, the instantaneous crack growth rate gives

(14) $$\Omega_{1\to 2}:da_{2}/dN_{2}=\alpha_{2}N_{1}^{\beta_{1}}=\alpha_{2}{\left[\frac{\alpha_{2}(\beta_{1}+1)}{\alpha_{1}(\beta_{2}+1)}\right]}^{\frac{\beta_{1}}{\beta_{1}+1}}\cdot N_{2}^{\frac{\beta_{1}(\beta_{2}+1)}{\beta_{1}+1}},$$

let $A=\alpha_{2}{\left[\frac{\alpha_{1}\left(\beta_{1}+1\right)}{\alpha_{2}\left(\beta_{2}+1\right)}\right]}^{\frac{\beta_{1}}{\beta_{1}+1}}$, $B=\frac{\beta_{1}\left(\beta_{2}+1\right)}{\beta_{1}+1}$ and the modified crack growth equation of BGA sample can be deduced as follows,

(15) $$\int_{0}^{a_{C}-a_{1}}da_{2}=\int_{0}^{N_{P_{1\to 2}}-N_{1}}A\cdot N_{2}^{B}dN_{2}\Rightarrow \Delta a=a_{C}-a_{1}=\frac{A}{B+1}{\left(N_{p}-N_{1}\right)}^{B+1},a_{C}=\frac{A}{B+1}{\left(N_{p_{1\to 2}}-N_{1}\right)}^{B+1}+\frac{\alpha_{1}}{\beta_{1}+1}N_{1}^{\beta_{1}+1}\Rightarrow N_{P_{1\to 2}}={\left[\frac{B+1}{A}\left(a_{C}-\frac{\alpha_{1}}{\beta_{1}+1}N_{1}^{\beta_{1}+1}\right)\right]}^{\frac{1}{B+1}}+N_{1},$$

finally, we work out the thermal fatigue lifetime prediction model:

(16) $$N_{f_{1\to 2}}=N_{o_{1}}+N_{p_{1\to 2}}=k_{1}{\left(\Delta W_{ave1}\right)}^{k_{2}}+N_{1}+{\left[\frac{B+1}{A}\left(a_{C}-\frac{\alpha_{1}}{\beta_{1}+1}N_{1}^{\beta_{1}+1}\right)\right]}^{\frac{1}{B+1}}.$$

### 2.4. Parameters Determination Based on Historical Crack Length Data

It is convenient that determining the values of α and β if the crack length data of a specimen in each load cycle is available. Take the logarithm of Equation (10), a relation between crack length and cycle number can be derived,

(17) $$\ln a=\left(\beta +1\right)\ln N+\ln\frac{\alpha }{\beta +1},$$

we can work out the least square estimates of α and β by

(18) $$\{\begin{array}{c} \hat{\beta }=\frac{\sum_{i=1}^{p}\ln N_{i}\ln a_{i}-p\bar{\ln N\ln a}}{\sum_{i=1}^{p}{\left(\ln N_{i}\right)}^{2}-p{\left(\bar{\ln N}\right)}^{2}}-1=\frac{\sum_{i=1}^{p}\left(\ln N_{i}-\bar{\ln N}\right)\left(\ln a_{i}-\bar{\ln a}\right)}{\sum_{i=1}^{p}{\left(\ln N_{i}-\bar{\ln N}\right)}^{2}}-1\\ \hat{\alpha }=\left(\hat{\beta }+1\right){\left(\prod_{i=1}^{p}\frac{a_{i}}{n_{i}^{\hat{\beta }+1}}\right)}^{1/p} \end{array},$$

However, these history data are deficient because these micro defects like crack and void are unobservable without destructive physical analysis (DPA) method, not to mention that this method cannot take sequence effects into consideration. Therefore, improved methods are urgently needed.

## 3. Discussion of Numerical Computation Method for Model Parameter Determination

In this paper, we propose a numerical computation method for determining parameters of a BGA life prediction model considering load sequence effects. This method is available without keeping a record of crack length, as long as the cycle numbers in several conditions are provided. It will be more convenient for forecasting the lifetime of BGA packaged products.

### 3.1. Parameters Determining Equations

In order to reflect the load sequence effects, more relations between load condition and life cycle need to be established. At least four equations are needed to determine all the parameters in $\Omega_{1}$ and $\Omega_{2}$ as Equation (19) shows.

(19) $$\{\begin{array}{l} \Omega_{1}:N_{f_{1}}=N_{o_{1}}+N_{p_{1}}=k_{1}{\left(\Delta W_{ave1}\right)}^{k_{2}}+{\left(\frac{\beta_{1}+1}{\alpha_{1}}\cdot a_{C}\right)}^{1/\left(\beta_{1}+1\right)}\\ \Omega_{2}:N_{f_{2}}=N_{o_{2}}+N_{p_{2}}=k_{1}{\left(\Delta W_{ave2}\right)}^{k_{2}}+{\left(\frac{\beta_{2}+1}{\alpha_{2}}\cdot a_{C}\right)}^{1/\left(\beta_{2}+1\right)}\\ \Omega_{1\to 2}:N_{f_{1\to 2}}=N_{o_{1}}+N_{p_{1\to 2}}=k_{1}{\left(\Delta W_{ave1}\right)}^{k_{2}}+N_{1}+{\left[\frac{B_{1}+1}{A_{1}}\left(a-\frac{\alpha_{1}}{\beta_{1}+1}n_{1}^{\beta_{1}+1}\right)\right]}^{\frac{1}{B_{1}+1}}\\ \Omega_{2\to 1}:N_{f_{2\to 1}}=N_{o_{2}}+N_{p_{2\to 1}}=k_{1}{\left(\Delta W_{ave2}\right)}^{k_{2}}+N_{2}+{\left[\frac{B_{2}+1}{A_{2}}\left(a-\frac{\alpha_{2}}{\beta_{2}+1}n_{2}^{\beta_{2}+1}\right)\right]}^{\frac{1}{B_{2}+1}} \end{array},$$

where $A_{1}=\alpha_{2}{\left[\frac{\alpha_{1}\left(\beta_{1}+1\right)}{\alpha_{2}\left(\beta_{2}+1\right)}\right]}^{\frac{\beta_{1}}{\beta_{1}+1}}$,$B_{1}=\frac{\beta_{1}\left(\beta_{2}+1\right)}{\beta_{1}+1}$,$A_{2}=\alpha_{1}{\left[\frac{\alpha_{2}\left(\beta_{2}+1\right)}{\alpha_{1}\left(\beta_{1}+1\right)}\right]}^{\frac{\beta_{2}}{\beta_{2}+1}}$,$B_{2}=\frac{\beta_{2}\left(\beta_{1}+1\right)}{\beta_{2}+1}$, the simplification of these simultaneous equations gives relations between $\beta_{1}$ and $\beta_{2}$, as Equation (20) shows,

(20) $$\{\begin{array}{c} {\left[\frac{1+\beta_{1}+\beta_{1}\left(\beta_{2}+1\right)}{\left(\beta_{2}+1\right)（\beta_{1}+1）}{\left(\frac{\beta_{2}+1}{\beta_{1}+1}\right)}^{\frac{2\beta_{1}}{\beta_{1}+1}}\left(1-{\left(\frac{n_{1}}{N_{p_{1}}}\right)}^{\beta_{1}+1}\right)N_{p_{1}}^{\beta_{1}}N_{p_{2}}^{-\frac{\beta_{1}(\beta_{2}+1)}{\beta_{1}+1}}\right]}^{\frac{1+\beta_{1}}{1+\beta_{1}+\beta_{1}\left(\beta_{2}+1\right)}}=N_{f_{1\to 2}}-N_{o_{1}}-n_{1}\\ {\left[\frac{1+\beta_{2}+\beta_{2}\left(\beta_{1}+1\right)}{\left(\beta_{1}+1\right)（\beta_{2}+1）}{\left(\frac{\beta_{1}+1}{\beta_{2}+1}\right)}^{\frac{2\beta_{2}}{\beta_{2}+1}}\left(1-{\left(\frac{n_{2}}{N_{p_{2}}}\right)}^{\beta_{2}+1}\right)N_{p_{2}}^{\beta_{2}}N_{p_{1}}^{-\frac{\beta_{2}(\beta_{1}+1)}{\beta_{2}+1}}\right]}^{\frac{1+\beta_{2}}{1+\beta_{2}+\beta_{2}\left(\beta_{1}+1\right)}}=N_{f_{2\to 1}}-N_{o_{2}}-n_{2} \end{array},$$

the approximate values of $\beta_{1}$ and $\beta_{2}$ can be worked out by Matlab software [19] with the test records of cycle numbers in different conditions. Afterwards, $\alpha_{1}$ and $\alpha_{2}$ can be calculated with Equation (21).

(21) $$\alpha_{i}=\frac{a\left(\beta_{i}+1\right)}{N_{p_{i}}^{\beta_{i}+1}},$$

### 3.2. Resistance Strain Theory

By nature of metal conductor resistance, according to solid fracture theory, the quantitative relationship can be revealed for the solder joint resistance strain and the crack expansion. The electrical resistivity of metal material increases as fatigue load cycles. Resistivity changes slightly in the initial crack stage, in the crack propagation stage resistivity varies relatively faster, when the material fails, fatigue damage mutates significantly as well as the resistivity [20].

Based on the Griffith fracture theory, the resistance strain of solder joints can be solved by Equation (22),

(22) $$\varepsilon_{R}=\frac{R_{t}-R_{0}}{R_{0}},$$

where $R_{0}$ is the solder joints resistances at time point $t_{0}$, and $R_{t}$ is the solder joints resistances at time point t.

**Figure 3:**
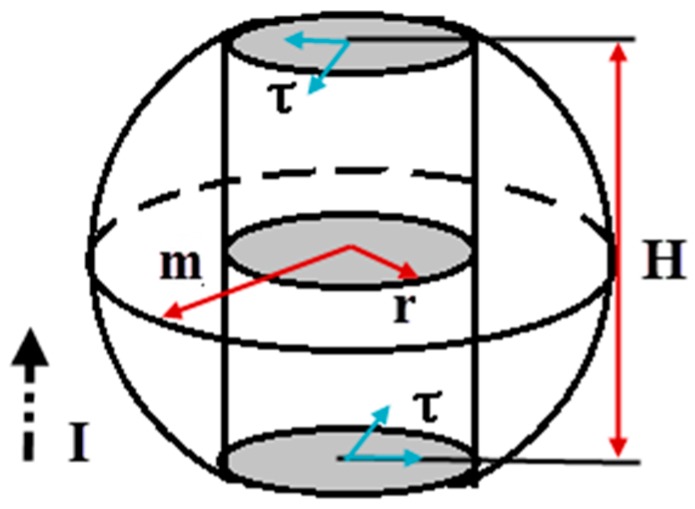
Schematic diagram of BGA solder joint and equivalent crack.

The geometry model of BGA solder joint is shown as Figure 3, I represents current direction passing the solder joint, τ represents the stress in the welding points. Supposing that only part of the damage crack defects, which are perpendicular to the current direction, have effects on the specimen resistance. When the solder joint is undamaged, its resistance can be solved by

(23) $$R_{0}=\rho \frac{H}{S},$$

ρ is material resistivity, H is solder joint thickness, S is the cross-sectional area that perpendicular to the current, and $S=\pi r^{2}$, *r* is the radius of welding points between solder point and PCB. When crack defects grow under load the resistance changes by

(24) $$R=\rho \frac{H}{S-S(a)}=\rho \frac{H}{\pi r^{2}-S(a)},$$

S(a) is a function of crack length, which represents the equivalent area of crack defects. Equation (24) indicates that when S(a)=0, the solder joint is undamaged and $R=R_{0}$. When S(a)<<S, means that crack is in initial stage, the resistance increases slowly and linearly dependent on the crack growth. The solder joint fractures when S(a) reaches the maximum value and resistance value tends to infinity nonlinearly (R→∞).

Differentiating Equation (24) with respect to all variables we obtain,

(25) $$dR=\frac{d\rho \cdot H}{\pi r^{2}-S(a)}+\rho \cdot \frac{dH}{\pi r^{2}-S(a)}+\rho H\cdot \frac{S^{\prime }(a)da}{{\left[\pi r^{2}-S(a)\right]}^{2}}-\rho H\cdot \frac{2\pi r\cdot dr}{{\left[\pi r^{2}-S(a)\right]}^{2}},$$

then

(26) $$\frac{dR}{R}=\frac{d\rho }{\rho }+\frac{dH}{H}+\frac{S^{\prime }(a)da}{\pi r^{2}-S(a)}-\frac{2\pi r\cdot dr}{\pi r^{2}-S(a)}.$$

In this paper, it is the thermal load conditions that dominate electrical resistance changes, and the influence of dr and dH can be neglected. So Equation (25) can be transformed into

(27) $$\varepsilon_{R}\approx \frac{\Delta R}{R}=\frac{\Delta \rho }{\rho }+\frac{S^{\prime }(a)\Delta a}{\pi r^{2}-S(a)}=\frac{\Delta \rho }{\rho }+\frac{\Delta S}{\pi r^{2}-S(a_{t})-\Delta S}+o(\Delta \rho \Delta S).$$

Assume that the equivalent area is a circle with radius a, thus $S(a_{t})=\pi a_{t}{}^{2}$, $a_{t}$ is the crack length at time point t, $S^{\prime }(a)=2\pi \Delta a^{2}$ and Δa is the increment of crack length in Δt. [21] points out that $\Delta \rho =\rho_{0}\times k\times \Delta T$, $\rho_{0}$ is the resistivity of material at a certain ambient temperature, k is the temperature coefficient of resistivity, ΔT is temperature range. Thus we have

(28) $$\varepsilon_{R}=\frac{\rho_{0}k\Delta T}{\rho_{0}(1+T_{1})}+\frac{2\Delta a^{2}}{r^{2}-a_{t}^{2}-2\Delta a^{2}}=\frac{k\Delta T}{\left(1+T_{1}\right)}+\frac{2\Delta a^{2}}{r^{2}-a_{t}^{2}-2\Delta a^{2}},a<r,$$

where $T_{1}$ is the initial temperature of the thermal cycle load.

Crack growth rate [22] can be expressed by $\frac{da}{dt}=m\times \omega^{\gamma }$, in which $m=8.34\times 10^{-8}$, γ=1.08, ω is related to the thermal stress inside of solder joint, in this paper we assume ω=0.003 which also follows the reference because of similar geometry and thermal conditions of solder joints. Combined with Equation (5) we have

(29) $$\{\begin{array}{c} a_{N}=a_{0}+C{\left(\Delta \sigma Y\sqrt{\pi a_{t-1}}\right)}^{n}\times N\\ a_{t}=a_{N}+m\times \omega^{\gamma }\times rem(t,t_{c}) \end{array},$$

With $rem(t,t_{c})$ means taking reminders of $t/t_{c}$, $t_{c}$ is the time that a single cycle may costs. Besides, parameters in the equation that *C* = 2.6 × 10^−13^, *n* = 2.1, *Y* = 1, and Δσ can be given by simulation which will be shown in following Section 3.3. As a result, the electrical resistance of solder joint in the (*N* + 1)th cycle is

(30) $$\varepsilon_{R}=\frac{k\Delta T}{\left(1+T_{1}\right)}+\frac{2{\left(m\times \omega^{\gamma }\times \Delta t\right)}^{2}}{r^{2}-{\left[a_{0}+C{\left(\Delta \sigma Y\sqrt{\pi a}\right)}^{n}\times N+m\times \omega^{\gamma }\times rem(t,t_{c})\right]}^{2}-2{\left(m\times \omega^{\gamma }\times \Delta t\right)}^{2}},$$

let *t_r_* represents the time of heating-cooling process, *t_s_* represents the holding time, $T_{L}$ represents the minimum temperature while *T_H_* means the maximum temperature, and the temperature changing rate is *b*. Equation (30) can be refined into

(31) $$\{\begin{array}{l} Heating:\varepsilon_{R}=\frac{kb(t-N\times t_{c})}{\left(1+kT_{L}\right)}+\nu \\ holding\_high:\varepsilon_{R}=\frac{k(T_{H}-T_{L})}{\left(1+kT_{L}\right)}+\nu \\ cooling:\varepsilon_{R}=\frac{kb[(N+1)\times t_{c}-t_{s}-t]}{\left(1+kT_{L}\right)}+\nu \\ holding\_low:\varepsilon_{R}=\nu \end{array},$$

in which

(32) $$\nu =\frac{2{\left(m\times \omega^{\gamma }\times \Delta t\right)}^{2}}{r^{2}-{\left[a_{0}+C{\left(\Delta \sigma Y\sqrt{\pi a}\right)}^{n}\times N+m\times \omega^{\gamma }\times rem(t,t_{c})\right]}^{2}-2{\left(m\times \omega^{\gamma }\times \Delta t\right)}^{2}}.$$

### 3.3. Stress Range Simulation

Mechanical behavior of solder joints under certain load profile can be computed by ANSYS software [23]. In order to analyze the stress and strain situation of BGA under different loading conditions and obtain the equivalent stress range mentioned in the previous section, simulation models of BGA specimens are constructed in software. To ensure the accuracy of simulation results, simulation models need to be in accord with the real specimen.

#### 3.3.1. Geometry Construction

**Figure 4:**
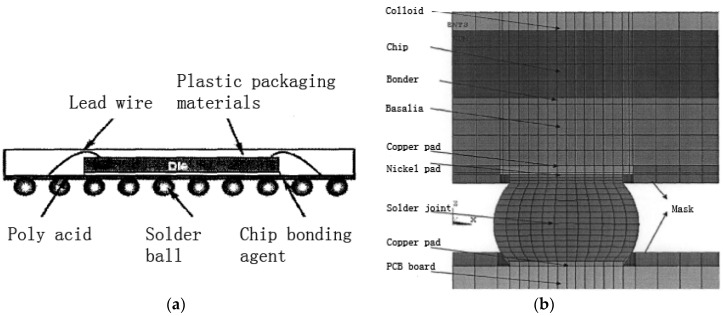
Structure diagram of MROM TF-BGA48: (**a**) Chip level structure; (**b**) Micro-structure of BGA solder joint.

In this paper MROM TF-BGA48 is selected as the research sample, the structure of which is as Figure 4 shows, detail size information is listed in Table 1.

**Figure 5:**
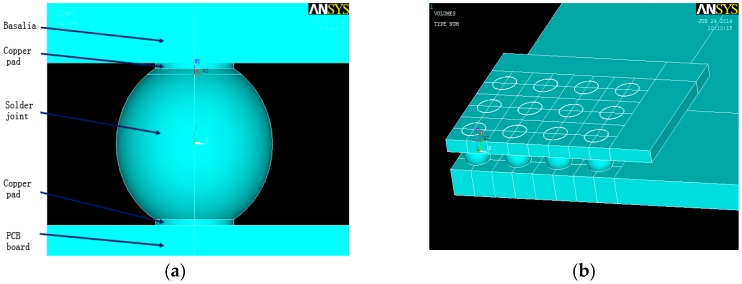
Simulation model of MROM TF-BGA48: (**a**) Single solder joint simulation model; (**b**) Chip level simulation model.

According to the size information, a simulation sample can be constructed in software, as Figure 5 shows.

#### 3.3.2. Initial Simulation Setting

For the purpose of describing the relationship between stress tensor and strain tensor, we can directly invoke a classical Anand constitutive model, which has been embedded into ANSYS. Furthermore, we chose Solid185 instead of Visoco107 for material unit in ANSYS, since the Solid185 can be used in simulating the deformation of more complex materials, such as incompressible elastic-plastic material or incompressible hyperelastic material, other material settings in the simulation are listed in Table 2. The full transient dynamic analysis method in software is used for large displacement analysis and the time increment of load substep values 10, other simulation settings like calculating times and convergence condition remain at defaults to ensure the convergence and accuracy of results.

#### 3.3.3. Mesh and Boundary Condition

**Figure 6:**
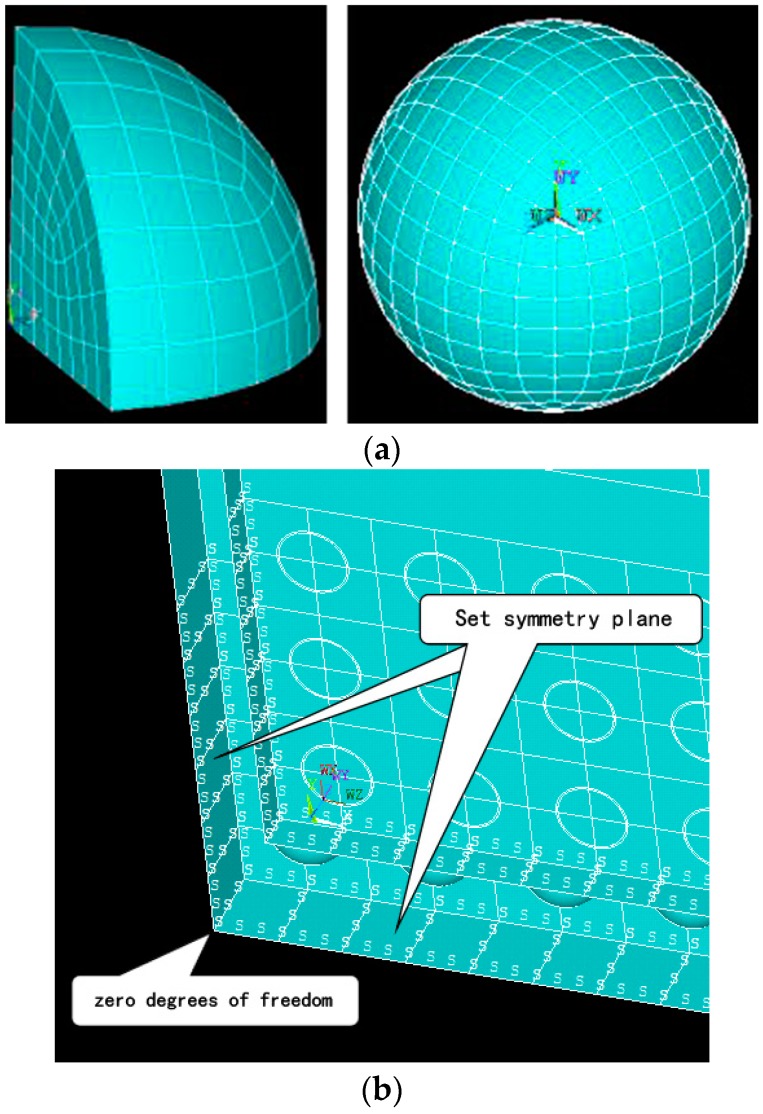
Mesh and boundary set in simulation model: (**a**) Meshes on welded ball of specimen; (**b**) Boundary conditions of symmetry plane and zero DOF.

Both the quantity and quality of mesh are significant to guarantee the accuracy of simulating results, especially the meshes on the welded ball of the specimen. Firstly, we divided a single welded ball model into quarters along two mutually perpendicular planes. Then we trisected the radius of a quarter of a welded ball, simultaneously quartering the remaining ball arc. After that, we executed the ‘sweep’ command to generate sufficient meshes, repeating the operation until the whole welded ball is meshed, as the Figure 6a shows.

To reflect the actual boundary condition of BGA specimens, symmetry plane constraint command is attached to the edge surfaces for prohibiting their displacements and rotary movements in simulation. Additionally, we set the PCB bottom point’s DOF (degree of freedom) as zero to avoid rigid body motion as Figure 6b shows.

#### 3.3.4. Thermal Load Profile

**Figure 7:**
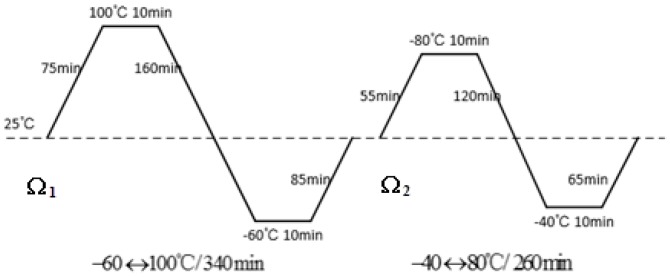
Thermal load profiles of $\Omega_{1}$ and $\Omega_{2}$.

The simulation thermal load profiles are set as Figure 7 shows. In $\Omega_{1}$, the maximum temperature is 100 °C and the minimum temperature is −60 °C, holding times are 10 min, the rate of temperature change is 1 °C/min. In $\Omega_{2}$, the maximum temperature is 80 °C and the minimum temperature is −40 °C, holding time and temperature changing rate are the same.

#### 3.3.5. Equivalent Stress Range

**Figure 8:**
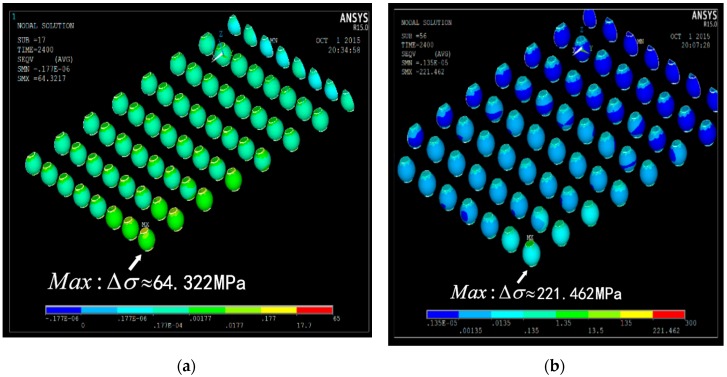
Distribution of stress and strain contours: (**a**) In $\Omega_{1}$ load profile; (**b**) In $\Omega_{2}$ load profile.

Figure 8 is the deformation nephogram of solder joints in BGA package structure under one of our thermal load conditions. It indicates the solder joints that are closer to the edge of package the larger plastic strain they will suffer, which coincides with the experiment results in [24]. Table 3 shows the simulation results of equivalent stress (Von Mises stress) range Δσ, plastic shear strain range Δγ, and fatigue life $N_{f}$. Default Coffin-Manson model is selected in ANSYS to give a preliminary fatigue life in $\Omega_{1}$ and $\Omega_{2}$, respectively.

### 3.4. Thermal Fatigue Life Computation

**Figure 9:**
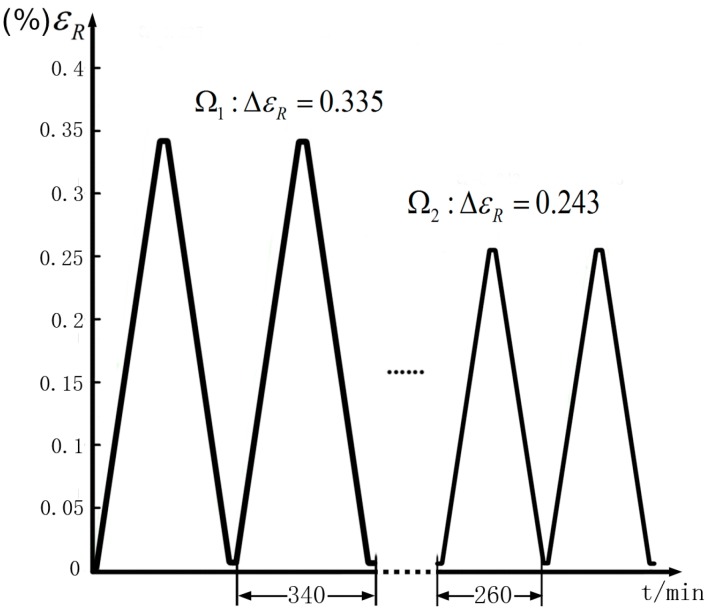
Resistance strain curve in certain thermal load conditions.

According to Equation (31), resistance strain curve under certain load conditions can be graphed as Figure 9 shows. The figure indicates that the variation of resistance strain is reversible, it increases or decreases as the temperature rises or falls during the thermal cycle. The amplitude of resistance strain variation depends on the temperature differences, the values of resistance strain increment are 0.335 and 0.243 in $\Omega_{1}$ and $\Omega_{2}$, respectively.

**Figure 10:**
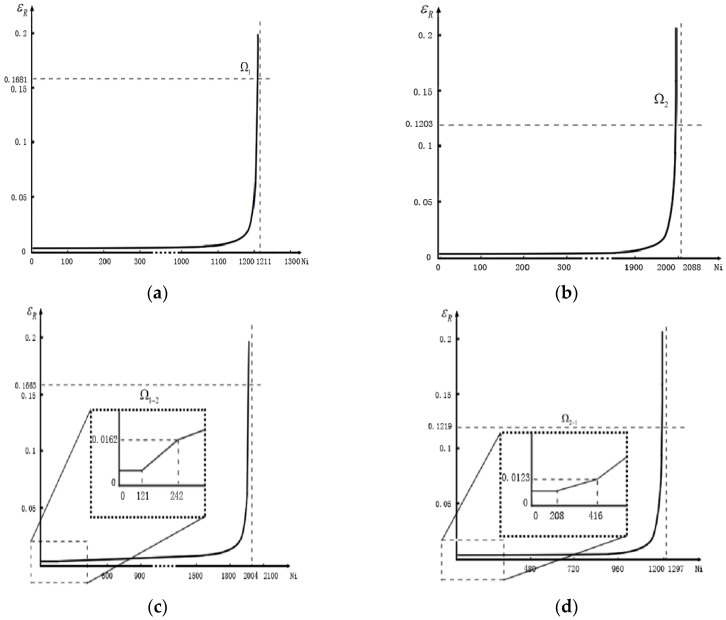
Relation between resistance strain and cycle numbers at T = 298 K under different load conditions: (**a**) $\Omega_{1}$; (**b**) $\Omega_{2}$; (**c**) $\Omega_{1\to 2}$; (**d**) $\Omega_{2\to 1}$.

The relationship between resistance strain and number of cycles can be obtained by extracting the information of resistance strain at certain temperature in each cycle. The relation curve under different thermal load conditions $\Omega_{1}$, $\Omega_{2}$, $\Omega_{1\to 2}$,$\Omega_{2\to 1}$ are graphed by programming in Matlab according to the theory described in Section 3.2 and the result are shown in Figure 10.

As (a) and (b) describe that there are some irreversible resistance strains during thermal cycle, this can be the results of irrecoverable defects like crack and void. The irrecoverable defects are negligibly small with respect to the recoverable defects in Figure 10, however, when these kinds of defects accumulate to some extent, a surge may occur in resistance strain. It turns out to be the consequence of crack length reaches the critical length $a_{C}$ (radius of solder points) that surge occurs at the same time. 

The simulation of thermal load sequence conditions $\Omega_{1\to 2}$ and $\Omega_{2\to 1}$ are based on the previous work. Solder joints spend their initial crack phase, which occupies 10% of the whole fatigue lifetime according to the fourth model assumption, under the first condition. We hypothesize that the specimen remains in the first condition until the next 1/10 of fatigue lifetime has passed, the load condition turns into the other condition afterwards. The simulation results showed in (c) and (d) indicate that a crack grows linearly before the surges. Moreover, the crack growth rate varies with the thermal load condition.

According to the simulation analysis, all information of fatigue life needed for calculating the life prediction model parameters are shown in Table 4.

**Figure 11:**
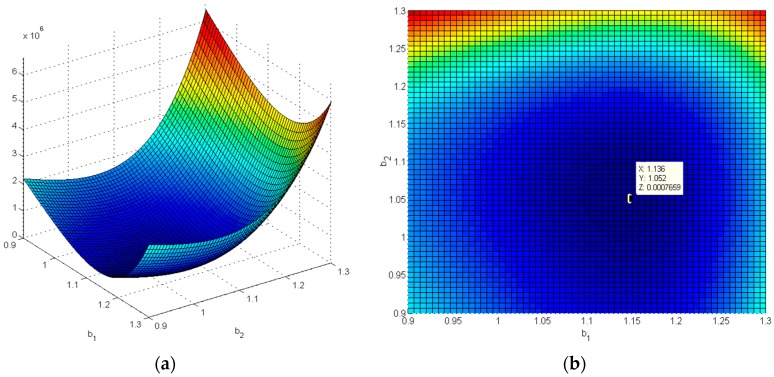
Simulation result of MATLAB: (**a**) 2-D parameter surface of $\beta_{1}$ and $\beta_{2}$; (**b**) Minimum point of $\beta_{1}$ and $\beta_{2}$.

Plug these fatigue data into Equation (19), the values of $\beta_{1}$ and $\beta_{2}$ can be solved by Matlab as Figure 11 shows, $\beta_{1}=1.136$ and $\beta_{2}=1.052$. So we can work out α_1_ = 7.8192 × 10^−9^ and α_2_ = 4.4132 × 10^−9^.

## 4. Experimental Verification

**Figure 12:**
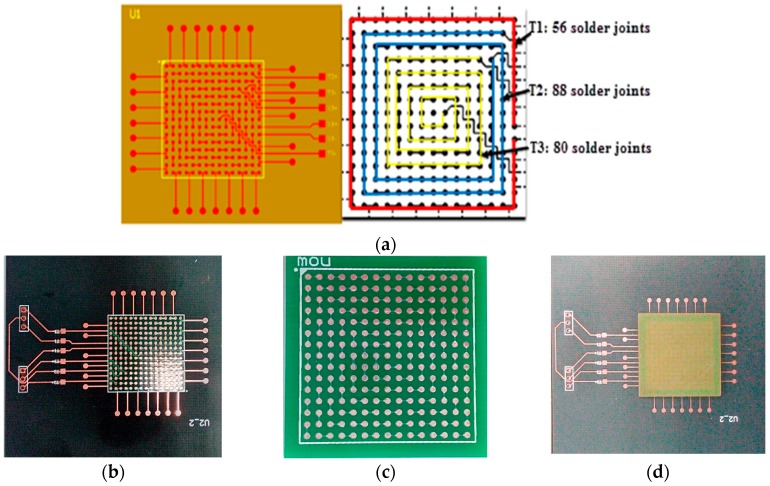
BGA package sample used in experiment: (**a**) Designed daisy chain structure; (**b**) Printed circuit board; (**c**) Chip substrate; (**d**) Welded sample.

Studies [25,26,27] demonstrate that daisy chain structure is an effective way to reduce the difficulty of monitoring a batch of solder joints. In daisy chain structure, severe damage in one single solder joint, especially the corners, often leads to the destruction of the entire package. The BGA package sample is designed as Figure 12 shows.

**Figure 13:**
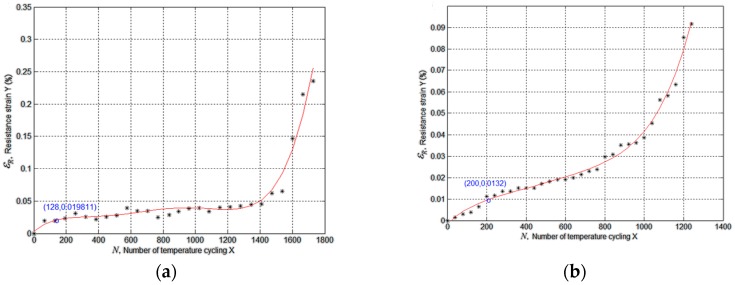

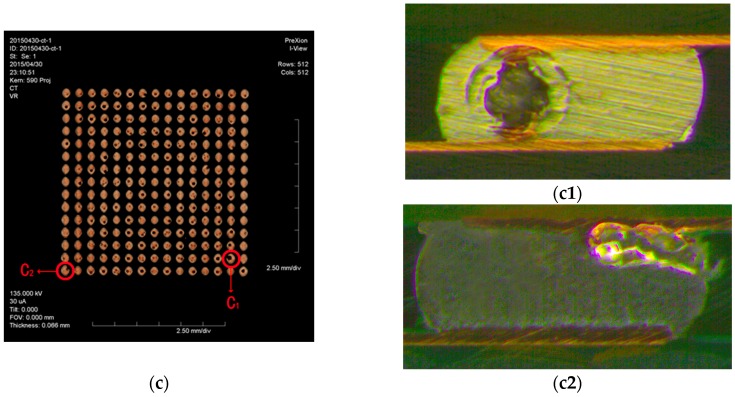
Relation curve between resistance strain and cycle numbers under: (**a**) $\Omega_{1\to 2}$ and (**b**) $\Omega_{2\to 1}$ condition of temperature cycle test; (**c**) Different crack appearances in solder joints at corner areas of the sample after test: (**c1**) crack penetrating into the solder joint; (**c2**) crack at the welded fringe.

Temperature cycle test is conducted following the $\Omega_{1\to 2}$ and $\Omega_{2\to 1}$ conditions, we record the resistances of solder joints in daisy chain at T = 298 K every five cycles. The relation curve between resistance strain and cycle numbers can be fitted by the records as shown in Figure 13a,b. After thermal cycle test, the SEM (scanning electron microscope) is used for location the crack defection region, and we observed that cracks arose a lot, especially at the corner solder joints as reference mentioned, as shown in Figure 13c.

According to Figure 13, we can see that in the early stage each condition the resistance strain grows slowly and linearly. The values of resistance strain at the condition changing time is similar to the simulation results, and the tendencies of $\varepsilon_{R}$ approximate to the results in Figure 10. In real experimental environment, fatigue is not the only factor that leads to damage, creep and other mechanisms may also exist. As a result, the experimental value of $\varepsilon_{R}$ is basically bigger than simulation results, and experiment life is shorter.

## 5. Conclusions 

Traditional fatigue prediction approaches are based on isothermal fatigue test data, but basically they cannot be appropriately used for engineering applications. Actually, most of the approaches ignore the load sequence influence and fatigue test data for mini-sized devices is hard to obtain, as a result of that, errors may be introduced if the data is not accurate.

In this article, we develop a new BGA life prediction model considering load sequence effects. Specifically, the analytic mathematic model is derived based on Darveaux model and Paris formula, whose parameters are determined by numerical simulation approaches. A resistance strain theory is proposed to reflect the crack growth progress by more convenient resistance data from fatigue test, and conducted by Mat lab software; the stress and strain situation that is needed for the theory is analyzed by modeling the BGA in ANSYS software. This work can help decrease the difficulty of getting fatigue test data effectively. Moreover, the resistance strain is discussed as microstructure damage related failure criteria and verified by experiment. However, the infinite element simulation of our model in ANSYS was able to analyze the stress and strain situation under different loading conditions, but cannot take the crack influence into consideration. Further research will be focused on improving the model, and our life prediction model may be a promising tool for future research in electronic device monitoring and life prediction.

## Figures and Tables

**Table 1:** Information of MROM TF-BGA48.

Model Components	Whole Package	PCB	Upper Copper Backing	Lower Copper Backing	Nickel Pad
(mm)	6 × 8 × 1.3	40 × 40 × 0.5	ϕ0.45 × 0.027	ϕ0.514 × 0.018	ϕ0.45 × 0.005
	**Welded-Spot pitch**	**Chip**	**Substrate**	**Binder**	**Colloid**
(mm)	0.8	4 × 5.6 × 0.254	6 × 8 × 0.2366	6 × 8 × 0.0254	6 × 8 × 0.53
	**Solder joint**				
	diameter (ϕ)	total height	upper cylinder height		material
(mm)	0.514	0.322	0.034		63Sn37Pb

**Table 2:** Material attribution setting.

Component Category	Elasticity Modulus (GPa)	Poisson’s Ratio	CTE (10^−6^/°C)
Solder joint	30	0.35	21
PCB (FR4)	48.2	0.3	19.17
Substrate	16.85	0.3	16
Copper backing	129	0.38	16.9
Specimen plate	17.4	0.15	15

**Table 3:** Simulation results under thermal load profiles.

Model Variables	$\Omega_{1}$	$\Omega_{2}$
Equivalent stress range Δσ (MPA)	221.462	64.322
Plastic shear strain range Δγ	0.03068	0.02334
Fatigue life $N_{f}$	1198	2045

**Table 4:** Fatigue life information.

Fatigue Variables	$\Omega_{1}$	$\Omega_{2}$	$\Omega_{1\to 2}$	$\Omega_{2\to 1}$
$N_{f_{i}}$	1211	2088	2004	1297
$N_{o_{i}}$	121	208	121	208
$N_{p_{i}}$	1090	1880	1762	881
$n_{i}$	/	/	121	208

## Nomenclature

$N_{f}$	the whole fatigue lifetime of solder joints, also the stress cycles of a specified character that a specimen sustains before failure of a specified nature occurs
$N_{f_{i}}$	the total fatigue life cycles of ball grid arrays under the *i*th thermal load condition
$N_{f_{i\to j}}$	the total fatigue life of ball grid arrays consists of certain fatigue cycles under the *i*th thermal condition and the rest cycles under the *j*th condition
$N_{o}$	the initial crack lifetime of solder joints, also the cycles to crack initiation
$N_{o_{i}}$	the initial crack lifetime of solder joints under the *i*th thermal condition
$N_{p}$	the crack propagation or growth lifetime of solder joints
$N_{p_{i}}$	the crack propagation lifetime of solder joints under the *i*th condition
$N_{p_{i\to j}}$	the crack propagation lifetime of ball grid arrays consists of certain propagation cycles under the *i*th thermal condition and the rest cycles under the *j*th condition
$N_{i}$	the actual cycles under the *i*th thermal condition, in which we assume that initial crack lifetime under *i*th thermal condition is included
$a,a_{C},a_{t},a_{N}$	crack length, critical crack length, crack length at certain time point *t*, and the crack length at certain cycle times *N*
$da_{i}/dN_{i}$	crack growth rate under the *i*th thermal load condition
$\Delta W_{avei}$	the incremental viscoplastic strain energy density under the *i*th thermal load condition
$\alpha_{i}$	constant related to thermal cyclic stress range Δσ in crack propagation stage under the *i*th thermal load condition
$\beta_{i}$	constant related the exponential increasing trend of crack growth ratio under the *i*th thermal load condition
$k_{1},k_{2},k_{3},k_{4}$	the correlation coefficients of material properties in Darveaux model
C,n	material constants of the Darveaux model in the Paris formula
ΔK	the amplitude of stress intensity factor
Δσ	the differences between the maximum stress value and minimum stress value under certain load conditions
Y	the geometry influence function whose value is related to the sample size and load form
$A_{1},A_{2},B_{1},B_{2}$	model parameters related with $\alpha_{i}$ and $\beta_{i}$
$\Omega_{i}$	the unchangeable load profile under *i*th thermal condition
$\Omega_{i\to j}$	the changeable load profile under *i*th condition then change to *j*th condition
$\varepsilon_{R}$	the resistance strain of solder joints
$\rho ,\rho_{0}$	the material resistivity and the resistivity of material at certain ambient temperatures
H	the thickness of solder joint
S	the cross-sectional area that perpendicular to the current, and $S=\pi r^{2}$
r	the radius of welding points between solder point and PCB
$t_{r},t_{s}$	the time of heating-cooling process and the holding time
$T_{H},T_{L}$	the minimum temperature and the maximum temperature
